# Consumers’ Concerns and Perceptions of Farm Animal Welfare

**DOI:** 10.3390/ani10030385

**Published:** 2020-02-27

**Authors:** Marta E. Alonso, José R. González-Montaña, Juan M. Lomillos

**Affiliations:** 1 Animal Production Department, Veterinary Faculty, University of León, Campus de Vegazana, 24071 León, Spain; 2 Medicine, Surgery and Anatomy Veterinary Department, Veterinary Faculty, University of León, Campus de Vegazana, 24071 León, Spain; jramirogonzalez@unileon.es; 3 Production and Health Animal, Public Health Veterinary and Science and Technology of Food Department, Veterinary Faculty, Cardenal Herrera-CEU University, C/Tirant lo Blanc, 7, 46115 Alfara del Patriarca, Valencia, Spain; juan.lomillos@uchceu.es

**Keywords:** animal welfare, consumer, concerns, farming, perceptions

## Abstract

**Simple Summary:**

In the current socioeconomic situation, consumers’ concerns about farm animal welfare can prevent them from buying some products, and this influences the sustainability of intensive systems. Consumers perceive the need to increase the level of welfare in farm animals, despite the fact that their level of knowledge about farming and animal welfare issues is relatively low. Consumers concerns are not equally distributed among the different farm species, nor is there consistency in the willingness to pay to enhance animal welfare. Therefore, farmers, businesses, and members of the food chain need to be informed of the evolution of public perceptions and consumer concerns to make informed decisions that help them improve their sustainability, social responsibility, and public credibility, which will allow them to maintain their social license through social ethical approval.

**Abstract:**

In this paper, we explore the evolution of consumers’ perceptions and concerns about the effects that intensification of production systems could have on the welfare of farm animals. Despite the differences in definitions of animal welfare that make perceptions about this complex subject extremely variable, there is a growing perception that farm animal welfare should be protected and improved. There is an increasing appreciation of animal welfare parameters over other quality attributes, and animal-friendly products are considered healthier, safer, tastier, more hygienic, authentic, environmentally friendly, and traditional by many consumers. The willingness to pay for the increases in price that higher levels of farm animal welfare could produce could be promoted by means of adequate information about management and housing conditions of the different farming species. Welfare-friendly products that are properly labeled with clear information provided by an internationally accepted, transparent, and traceable monitoring system will increase consumers’ confidence in the food chain participants. Both consumers and citizens have the opportunity to improve the welfare of millions of farmed animals now and in the future, consumers by assuming their responsibility at the buying point, purchasing welfare-friendly products, and citizens by driving legislation to achieve some minimum standard of welfare conditions that could meet animals’ needs.

## 1. Introduction

Over the last fifty years, the livestock sector has undergone changes without precedents to deal with the increasing demand of food derived from animals in the world’s most rapidly growing economies [1]. The global human population increased by a factor of 2.4 and meat consumption by a factor of 4.7 in this period [2]. More efficiency [3] and intensification of livestock production systems seems inevitable [4] as the only possibility to meet the 72 kg global meat consumption increase per head/per year expected until 2050 [5]. A rise in income per capita traditionally increases the consumption of foods with higher a content of animal protein, fats, and sugars [6], moving industrialized and developing countries towards similar consumption patterns of animal products [7]. The technological innovations and structural transformations of the livestock sector offers opportunities for poverty reduction and an increase in food security [1], but there are different issues that should be addressed to make the systems more sustainable. In this sense, a wider concept of sustainability appeared in relation to resource availability as a consequence of function and morality of action [8]. Inefficient usage of world natural resources, adverse human health and welfare effects, harmful environmental consequences, and poor welfare of animals could make a system or procedure unsustainable because members of the public consider them unacceptable [9].

In the current socioeconomic global situation, there is strong evidence of public concern over the moral implications of actual animal production systems on farm animal welfare [2,10,11]. As proof that the animal-ethics dimension of sustainability is considered [12,13], the United Nations Committee on World Food Security stated in its ‘Proposed draft recommendations on sustainable agricultural development for food security and nutrition including the role of livestock’, Recommendation ‘D’ of Article VIII, entitled ‘Animal health and welfare’: “*Improve animal welfare delivering on the five freedoms and related OIE standards and principles, including through capacity building programs, and supporting voluntary actions in the livestock sector to improve animal welfare*” [4,14] (OIE stands for Organization for Animal Health). In 2014, the International Finance Corporation (IFC) published a Good Practice Note entitled Animal Welfare in Livestock Operations, which clearly pointed out that “*Higher animal welfare standards are increasingly seen to be a prerequisite to enhancing business efficiency and profitability, satisfying international markets and meeting consumer expectations*” [15]. The IFC policies are applied throughout the world [8].

Public concern about animals used by humans is not a new phenomenon, and it increased in importance during the last century, beginning with the British animal protection legislation of the nineteenth century [4], continuing with the publication of Ruth Harrison´s book Animal Machines in 1964, followed by the development of philosophical arguments defending animal welfare [16] and animal rights [17]. Now, it has reached a point where the poor welfare of animals is a major reason why the public consider some animal production systems as unacceptable [18]; intensive animal production systems raise public criticism regarding how they affect animal welfare [19].

Over the last two decades, increasing numbers of consumers and citizens demanded ethical production systems and claimed to refuse to buy products that did not meet their animal welfare concerns [8]. In Europe, comparing the results of the surveys of 2006 and 2015 [10,11], an increasing concern was expressed. Respondents in 2006 rated 7.8/10, on average, the level of importance that they personally assigned to the protection of farmed animal welfare [10]. The percentage of citizens that considered that it is very important to protect the welfare of farmed animals increased from 34% to 57% between 2006 and 2015 [10,11]. Different studies have documented that the situation is very similar worldwide (EU [20,21,22,23], USA [24,25,26,27], Canada [28,29], Latin America [30,31,32,33], Asia [34], and Australia [35]), demonstrating that animal welfare concerns have become more important over the years, as was predicted by the authors of [36].

To address the farm animal welfare issue is not simple, and it is considered by some authors as a “wicked problem” [2] since it involves many aspects of a complex nature and many stakeholders’ have different interests in relation to decisions and policymaking [37].

## 2. Farm Animal Welfare: A “Complex Problem”

One of the first problems we face when we aim to compare studies and scientific papers on perspectives and concerns of citizens and consumers is the lack of a globally accepted definition of animal welfare [38]; this term is used with varied meanings [39], and different stakeholders have different definitions and perceptions of animal welfare [40,41]. Over the last five decades, aspects of the animal’s ability to cope with the environment [42,43], quality of life experienced and evaluated by the animal [44], and positive and negative mental states [45,46,47] produced a variety of definitions depending on the degree of importance of the aspects considered. Fraser [48] classified the scientific approaches to define animal welfare into three groups depending on whether they focused on the biological functioning of the animal (objective); the emotions experienced by the animals or affective state (subjective); and, finally, if the behavior or environment in with the animal lives is similar to those of the natural state of the species (natural living). Despite the discrepancies, as this is a very complex concept [49], there are some points of consensus, for example, that welfare is an inherent characteristic of the animal and not of the environment [50] and welfare varies over time on a continuous scale from very bad to very good, necessitating the use of different scientific measures to assess it [51] because it is a multidimensional concept [52].

The Five Freedoms, initially proposed by the Brambell Committee in 1965 and refined in 1979 by the UK’s Farm Animal Welfare Council (FAWC) have been used internationally as the conceptual framework to describe fundamental principles of animal welfare [53]. More recently, the Welfare Quality Project (WQP), funded by the European Commission, identified 12 criteria for the assessment of animal welfare that complement the Five Freedoms approach. On the other hand, the Five Domains expanded the Five Freedoms’ basic principles of animal welfare needs, focusing on physical and functional welfare in the domains of nutrition, environment, health, behavior, and mental state [54]. Finally, in the “A Life Worth Living” definition, animal welfare is the negative or positive quality of life observed in animals, and it depends on how they feel at a particular time and place [55]. All these different approaches produced a systematic disagreement between lay and expert views about what is a good animal life [56].

The definition of animal welfare provided by the Organization for Animal Health (OIE) includes some of the different points mentioned above and considers “… *how an animal is coping with the conditions in which it lives. An animal is in a good state of welfare if (as indicated by scientific evidence) it is healthy, comfortable, well nourished, safe, able to express innate behaviour, and if it is not suffering from unpleasant states such as pain, fear, and distress*”. Through the introduction of this definition in the OIE Terrestrial Animal Health Code, this international organization recognizes the importance and priority of animal welfare [38]. This code is the principal source of international standards on animal health and recommendations on animal welfare for farm animals, and its recommendations have a scientific basis (Chapter 7.1 art. 7.1.3) [53].

The relevance of the lack of a globally accepted definition is based on the fact that depending on which of the three views of this concept is considered predominant (biological functioning, affective state, or natural living [48]), the parameters designed and used to assess whether the welfare of an animal is good or poor are different, and these measures of welfare are valuable to use in legislation and standards [8]. As Fraser [48] wisely writes “*It would be comforting to think that science could simply set things straight by replacing these different, value-dependent views of animal welfare with objective data about what is truly better for animals*”. A solid, scientific foundation would reinforce the connection between animal welfare and animal health and help to develop animal welfare legislation that could cope with public concerns from a scientific approach and not be based on "common sense" or the tendency to equate "traditional" or "natural" husbandry practices with animal welfare [53]. This is necessary to develop a dynamic and strong relationship between animal welfare scientists and regulatory agencies to improve animal welfare legislation. In order to keep legislation in line with scientific developments, principal national legislation should be kept simple, with the more detailed requirements set out in implementing regulations and other subsidiary legislation that can more easily be changed [53].

Making the situation of different definitions of animal welfare even more complicated, which in turn could hinder the development of a straightforward measurement and assessment of animal welfare [49], some papers claims that moral and ethical society standards should be considered because of the values laid on its animals by society [57]. However, the study of animal welfare cannot be based entirely on science due to differences in ethical assumptions and potential conflicts of view that need to be to be recognized and debated [56,58]. Animal welfare should not be understood as a simple additive function of negative or positive states [59], and the existence of diverse concepts of nature related to welfare, culturally and historically, suggests the need for further research [60].

## 3. Citizens’ and Consumers’ Concerns and Attitudes

First, we should start differentiating the roles of consumers and citizens in relation to the moral implications of farm animal welfare. Citizens participate in the process of public opinion formation [61], drive legislation, and have influence on political decisions made by the governments voicing their public concerns for farm animal welfare [38,62,63]. Voting, writing letters to politicians and media, and participation in associations are some of the common citizen behaviors [50,61]. Changing attitudes, behaviors, and opinions could form an important driving force for improvements in the ethical status of farm animals in society [23]. Consumers, on the other hand, have an influence in the marketplace because they can change their buying behavior or refuse to buy products from systems with some aspects they do not like [42]. We will explore later the degree of expression of consumers’ attitudes on shopping behavior, but consumers also have a role as citizens, and during the beginning of the twenty-first century, the channeling of concerns about some animal products was mainly through the citizen role [61].

Sometimes the terms concern and attitude are used indistinctly, but attitude is a psychological tendency that is expressed by evaluating a particular entity with some degree of favor or disfavor [64] and concern is consider the evaluation of or an attitude towards some issue [65]. Following Brom [66], we consider that it is important to distinguish between three types of concerns. In the first group there are concerns that matter equally to everybody in their role as consumers. In different studies across the world safety is a primary concern for all food consumers, and at the end of twentieth century and beginning of the twenty-first century, this was the highest concern due to some food scandals involving, for example, Bovine Spongiform Encephalopathy (BSE), salmonella, and dioxins [67,68].

In the second group are concerns that matter to special groups of consumers, because it is important for them to be able to live according to their own life plan. Even when, as Ajzen [69] pointed out, attitudes are not always translated into intentions and the intentions may not be translated into performance or action, some people do act or want to act according to their attitude. The difficulties to translate concerns and attitudes about farm animal welfare into buying behavior could be one of the causes that explains why sales may be different from stated preferences or attitudes [8].

The third group of concerns matter to people in their role as citizens and are related to ideas about a good society. They are public moral concerns not “consumer concerns” in a technical sense. People could be concerned about certain products because of the wider impact they have on their society and the world, for example, excessive use of natural resources or environmental damage that could make the production system ethically unsustainable [9].

Both consumers and citizen have the opportunity to improve their society by achieving higher levels of animal welfare, which are considered, in Europe and around the world, to be part of a public good in their own right [70,71]. Farming activities are no longer viewed as simply a means of food production, but are fundamental to other key social goals such as food safety and quality, safeguarding environmental protection, sustainability, and enhancing the quality of life in rural areas [72].

As previously mentioned, in the case of Europe, citizens expressed a high degree of concern in the Eurobarometer surveys of 2006 [10] with 34% of surveyed people reporting to be strongly concerned about animal welfare. In a study carried out in the UK during the same period, only 20% of consumer respondents were strongly concerned about animal welfare [73]. The Eurobarometer results in 2016 showed 57% of EU citizens expressing a strong concern about animal welfare compared with 78% of the UK respondents [11]. These data are in accordance with Miele [74], who reported a 73% general interest in animal welfare of citizens, but only 39% thinking of animal welfare when buying meat, in a survey made in the UK and six other countries as part of the Welfare Quality Project. This supports the existence of a discrepancy between an individual’s role as a citizen and as a consumer [61,75], showing different concerns in different contexts.

Some people may express more concerns or preferences for systems that provide higher animal welfare standards when interviewed [22] but not at the purchasing point because they consider other product attributes and price as priorities [76,77]. On the other hand, the social desirability bias due to the fact that positive attitudes towards animal welfare are considered ethically virtuous could have some influence over self-reported concerns [21,63].

Finally, citizens include groups of people who never or very seldomly buy products of animal origin, such as vegans and vegetarians. They hold strong positions and are active members of society [2] and want to improve the animal welfare of all animals including farmed ones.

## 4. Determinants of Consumers’ Concerns

It is important to elucidate and understand the causes that underpin the global consumers’ farm animal welfare concerns. Firstly, one potential explanation is that consumers consider farm animal welfare as an attribute of the food quality concept [72] with increasing importance over other attributes [61,78,79,80,81]. In this sense, in 2001, Harper and Henson [68] reported that consumers of the United Kingdom, Ireland, France, Germany, and Italy were concerned about food safety, health, and quality, but they did not prioritize farm animal welfare over other food concerns. They used animal welfare as an indicator of other attributes associated with human health and safety. A diminution in the consumption of foods from animal origin during the late twentieth and early twenty-first century was attributed mainly to consumers’ beliefs about the negative influence on health due to problems caused by BSE, salmonella, and dioxins and safety-related issues [20,26,36,67,68]. There is an association between farm animal welfare and higher human health benefits, and this is one of the main reasons why people prefer to buy animal-welfare-friendly products [30,35,63,72,82,83,84,85,86,87,88]. Organic production systems are also viewed by consumers as more welfare friendly, with higher standards of farm animal welfare than conventional livestock systems, and better for human health due to low or no use of chemicals [68,81,89,90]. This would indicate that when rating food attributes, safety and individual benefits are more highly rated than societal or animal benefits per se [30,88,89].

However, animal-friendly products are not only perceived by consumers as healthier, they are also considered of higher quality, tastier, more hygienic, safer, acceptable, authentic, environmentally friendly, and traditional [63,73,83,91,92]. On the other hand, consumers also perceive that farming conditions that negatively affect animal welfare could also damage other quality aspects [72]. In this regard, the perceived quality of products from systems with higher levels of farm animal welfare is high as the quality of food definition relates to being fit for human consumption and its ability to satisfy stated or implied needs [93]. This seems to be corroborated by empirical literature showing high food quality is correlated with high levels of animal welfare [90,94,95,96]. 

Another important finding is that negative consumer attitudes and concerns towards intensive production systems [21,62] are not equally distributed among the different farming species [20]. Farming conditions of broilers and layer hens are considered the worst in relation to farm animal welfare, with a more positive perception of cattle conditions and pig production, with some variations among studies [20,68]. There is a duality in concerns expressed and the reduction in animal products consumption because in some studies it was reported that the consumption of beef and pork was more likely to decrease and that chicken consumption was more likely to increase with some variations among countries, although these trends could also be due to health and safety problems, as mentioned above, not just welfare considerations [20,26,36,68]. At present, the trend for meat consumption seems to be increasing [5], and it may be that some of the ethical farm-animal-welfare concerns are reduced with the enforcement of updated legislation to reflect public concerns [97], private retail company codes of practice for animal production, or the inclusion of farm animal welfare in corporate social responsibility schemes [98]. In Europe, in particular, this may not be the main reason because, despite the important changes in the European legislation controlling livestock industries, 82% of respondents of the Eurobarometer 2016 survey thought that farm animal welfare should be better protected than it was at the time the survey was made, and 89% thought that there should be a law obliging any person using animals for commercial purposes in the EU to care for them [8,11].

The differences in concerns relating to different animal species are also reflected in the willingness to pay for welfare-friendly products, from least to greatest: pigs, fish, broiler chickens, layer hens, dairy and beef cows, according with the [62] meta-analysis. The low willingness to pay for improvements in welfare in pig farming is surprising and could be due to the fact that in some countries the consumers consider that improvements should be made by legislation enforcement and not by premium prices [99]. Another reason could be that consumers consider other pork meat attributes more relevant than farm animal welfare at purchase [61].

Attitudes and willingness to pay for welfare-friendly products also differed with socio-demographic characteristics, with a high degree of consensus among the reviewed literature supporting that women, younger participants, pets owners, and those who had spent longer in education and had higher income rates demonstrated the highest levels of concern and were most likely to be willing to pay for welfare-friendly products [20,21,22,23,26,30,31,33,34,35,62,68,80,99,100,101,102,103]. A study carried out in China reported no difference in gender, which may be explained by a deep-rooted cultural concept that animals should be respected as an essential part of society [34]. For a thorough review of these factors, see Cornish´s 2016 work [38]. Of particular relevance is the fact that younger citizens and consumers are more concerned and more willing to pay for welfare-friendly products, and they will be the main drivers of the food market in the future. 

There is a discrepancy between the results of self-reported public concerns about farm animal welfare and the willingness to pay for welfare-friendly products, because the increase in the first is nor reflected in the second, as only marginal or small price premium increases for farm animal welfare, in some cases not enough to cover the costs, are reported in some studies [62,99,104,105,106,107,108]. In Europe, only a minority (3%) is ready to pay increases more than 20%, and 35% are not ready to pay any increases [11]. It is possible that for some consumers their attitudes are weak and do not affect their purchasing behavior or willingness to pay premium prices [61]. In other studies, a dissociation between live animals and the food they produce was interpreted as the cause for not choosing welfare-friendly products [109]. However, in any case, there is a positive desire to improve farm animal welfare, regardless of the type of animal or welfare issue being considered [62]. Therefore, it would be desirable to take a global approach to animals’ needs in terms of welfare, and different aspects of management, such as housing conditions, the environment, and transport, should be considered in the political decision-making process [62]. 

At this point, we should consider that consumers´ concerns and willingness to pay for products from systems with high levels of farm-animal welfare co-exist with low levels of knowledge about farming issues in general and animal welfare in particular [10,35,36,61,73,77,86,106,110,111]. This could be due to the growing mental and physical distance between consumers and producers [66,111], lack of involvement of urban consumers in livestock matters [81], information derived from mass media that focused predominantly on negative issues [68,74] or was provided by animal protectionists such as the Humane Society of the United States (HSUS) and People for the Ethical Treatment of Animals (PETA) [26], no firsthand experience [112], and disconnection in communication around animal welfare between consumers and producers [24,41]. All of these factors may have led to the current situation in which consumers’ farm-animal-welfare views may be driven less by facts and more by perceptions (or misperceptions) [35].

For years, food experts, animal protection organizations, consumer organizations, and food policy authorities have been the sources of animal welfare information for European consumers [113]. One of the best ways to increase relevant knowledge is through farm visits, because this would allow consumers to experience and learn about what farming entails and how farm animals live [112], but some people do not like to think about the link between live animals and food products when making purchasing decisions [68,114,115,116,117] as a disassociation psychological strategy as a way to deal with the meat paradox [20,68,118]. In this situation it is important to provide information so that it does not increase the disassociation [68,117,118].

Consumers’ self-reported willingness to increase knowledge about farming and animal welfare [11,30,33,62,81,109,119] could be addressed by labeling schemes with clear, rational, scientifically-based, and comprehensible information [68], increasing transparency and confidence in the food chain participants [79,120]. Labeling information could also help consumers to assume their political responsibility as participants in a free market [66,73] and make informed buying decisions [121], becoming “ethically competent consumers” [71]. However, some consumers may not want to accept this responsibility [8,20,71] and prefer to delegate it to governments or other participants in the food chain [8,49,122], or they may declare they cannot handle all the information of food labels as they are too complex [20] or they do not trust the information that is presented [105].

An increase in the level of consumers’ education through an appropriate methodology [123] is desirable because it is correlated with the level of moral concerns and willingness to pay for welfare-friendly products [36,73,101,124,125,126]. Food labels that identify welfare-friendly products seem to be an appropriate tool as more than half (52%) of the European respondents used them to identify welfare-friendly products [8,11]. In order to maximize the utility of the labeling system, it should be based on standardized indicators, scientifically developed and recognized in the EU and internationally [72], and provide a transparent and traceable monitoring system for animal-welfare-friendly products [120]. So far, there is no such system due to the lack of an international consensus about a universally accepted standard of animal welfare and the role of animal welfare in production systems [4]. The International Standard Organization ISO TS 34,700 published in 2016 could be a framework for voluntary adoption if no other animal welfare assurance schemes or standards legislations are developed [4].

Farmers, companies, and food chain members should be aware of public perceptions and the evolution of consumers’ concerns and attitudes so as to make informed decisions regarding the implementation of production practices on their farms that enhance sustainability, social responsibility [26], and public trust, while maintaining their social license [27] through ethical approval by society in general [63]. The strategies to meet the consumers’ requirements could actually be a business opportunity, allowing the farmers to continue operating in a more welfare-friendly sustainable manner and still be economically profitable [72]. In the same way, from a psychological point of view, farmers will benefit because they do not like to be seen as incompetent or uncaring to animals under their stewardship by the public due to media coverage of farm-animal-welfare issues [8].

## 5. Final Remarks

Despite the differences in the concepts and definitions of animal welfare that make perceptions about this subject very variable, over the last few years there has been a growing concern among citizens and consumers about the effects that the intensification of animal production systems could have on the welfare of farm animals. There is an increasing appreciation of animal welfare parameters over other quality attributes of food products. Animal-friendly products are considered healthier, tastier, more hygienic, safer, acceptable, authentic, environmentally friendly, and traditional by consumers.

The willingness to pay for the increases in price that higher levels of farm animal welfare could require should be promoted by means of adequate information about management and housing conditions of the different farming species. Welfare-friendly products should be properly labeled with clear information provided by an internationally accepted, transparent, and traceable monitoring system, increasing consumers’ confidence in the food chain participants.

Both consumers and citizens have the opportunity to improve the welfare of millions of farmed animals now and in the future, consumers by assuming their responsibility at the buying point, purchasing welfare-friendly products, and citizens by driving legislation to achieve some minimum standard of welfare conditions that could meet the animals’ needs. The policy and legal requirements of some countries could also improve the animal welfare in other countries that do not have this legislation but trade and want to sell their products in the former.

Improving the farming systems to achieve animals living in conditions of good health and welfare could reduce the incidence of diseases and the use of antibiotics as part to a strategy to diminish the global bacterial resistances, and this would result in better human health and welfare, all linked in the One Health One Welfare goal.

## References

[1] FAO Animal Production. FAO’s Role in Animal Production. http://www.fao.org/animal-production/en/.

[2] Fernandes J., Blache D., Maloney S.K., Martin G.B., Venus B., Walker F.R., Head B., Tilbrook A. (2019). Addressing animal welfare through collaborative stakeholder networks. Agriculture.

[3] Godfray H.C.J., Garnett T. (2014). Food security and sustainable intensification. Phil. Trans. R. Soc. B.

[4] Buller H., Blokhuis H., Jensen P., Keeling L. (2018). Towards farm animal welfare and sustainability. Animals.

[5] Miele M. Public attitudes and understanding of animal welfare standards: Could one welfare help? Animal welfare for a better world. Proceedings of the 4th OIE Global Conference on Animal Welfare.

[6] Grigg D. (1995). The pattern of world protein consumption. Geoforum.

[7] Allievi F., Vinnari M., Luukkanen J. (2015). Meat consumption and production—Analysis of efficiency, sufficiency and consistency of global trends. J. Clean. Prod..

[8] Broom D.M. (2017). Animal Welfare in the European Union.

[9] Broom D.M. (2019). Land and water usage in beef production systems. Animals.

[10] European-Commission (2007). Attitudes of EU Citizens towards Animal Welfare, Report.

[11] European-Commission (2016). Attitudes of EU Citizens towards Animal Welfare, Report.

[12] Rawles K., D’Silva J., Webster J. (2010). Developing ethical, sustainable and compassionate food policies. The Meat Crisis: Developing More Sustainable and Ethical Production and Consumption.

[13] Vinnari M., Vinnari E. (2014). A framework for sustainability transition: The case of plant-based diets. J. Agric. Environ. Ethics.

[14] United-Nations (2016). Sustainable Agricultural Development for Food Security and Nutrition: What Roles for Livestock? A Report by the CFS High Level Panel of Experts on Food Security and Nutrition.

[15] International Finance Corporation (2014). IFC Good Practice Note: Improving Animal Welfare in Livestock Operations.

[16] Singer P. (1975). Animal Liberation: A New Ethics for Our Treatment of Animals.

[17] Regan T., Singer P. (1985). The case for animal rights. Defense of Animals.

[18] Ryan Y.M. (1997). Meat avoidance and body weight concerns: Nutritional implications for teenage girls. Proc. Nutr. Soc..

[19] Appleby M.C. (1999). What Should We Do about Animal Welfare?.

[20] Clark B., Stewart G.B., Panzone L.A., Kyriazakis I., Frewer L.J. (2016). A systematic review of public attitudes, perceptions and behaviours towards production diseases associated with farm animal welfare. J. Agric. Environ. Ethics.

[21] Maria G.A. (2006). Public perception of farm animal welfare in Spain. Livest. Sci..

[22] Vanhonacker F., Verbeke W., Van Poucke E., Tuyttens F. (2007). Segmentation based on consumers’ perceived importance and attitude toward farm animal welfare. Int. J. Sociol. Agric. Food.

[23] Kupsala S., Vinnari M., Jokinen P., Rasanen P. (2015). Citizen attitudes to farm animals in Finland: A population-based study. J. Agric. Environ. Ethics.

[24] Kendall H.A., Lobao L.M., Sharp J.S. (2006). Public concern with animal well-being: Place, social structural location, and individual experience. Rural Sociol..

[25] Prickett R.W., Norwood F.B., Lusk J.L. (2010). Consumer preferences for farm animal welfare: Results from a telephone survey of US households. Anim. Welf..

[26] McKendree M.G.S., Croney C.C., Widmar N.J.O. (2014). Effects of demographic factors and information sources on United States consumer perceptions of animal welfare. J. Anim. Sci..

[27] Wolf C.A., Tonsor G.T., McKendree M.G.S., Thomson D.U., Swanson J.C. (2016). Public and farmer perceptions of dairy cattle welfare in the United States. J. Dairy Sci..

[28] Bejaei M., Wiseman K., Cheng K.M. (2011). Influences of demographic characteristics, attitudes, and preferences of consumers on table egg consumption in British Columbia, Canada. Poult. Sci..

[29] Spooner J.M., Schuppli C.A., Fraser D. (2014). Attitudes of Canadian citizens toward farm animal welfare: A qualitative study. Livest. Sci..

[30] Miranda de la Lama G.C., Estevez-Moreno L.X., Sepulveda W.S., Estrada Chavero M.C., Rayas Amor A.A., Villarroel M., Maria G.A. (2017). Mexican consumers’ perceptions and attitudes towards farm animal welfare and willingness to pay for welfare friendly meat products. Meat Sci..

[31] Schnettler B., Vidal R., Silva R., Vallejos L., Sepulveda N. (2008). Consumer perception of animal welfare and livestock production in the Araucania Region, Chile. Chil. J. Agric. Res..

[32] Santurtun Oliveros E., Tapia Perez G., Gonzalez Rebeles C., Galindo Maldonado F. (2012). Consumer attitudes and perceptions towards sustainable animal production attributes in Mexico City. Vet. Mexico.

[33] Vargas Bello Perez E., Riveros J.L., Kobrich C., Alvarez Melo P.A., Lensink J. (2017). Chilean consumers’ perception about animal welfare in dairy production systems: Short communication. Anim. Prod. Sci..

[34] Su B., Martens P. (2017). Public attitudes toward animals and the influential factors in contemporary China. Anim. Welf..

[35] Malek L., Umberger W.J., Rolfe J. (2018). Segmentation of Australian meat consumers on the basis of attitudes regarding farm animal welfare and the environmental impact of meat production. Anim. Prod. Sci..

[36] Ellis K.A., Billington K., McNeil B., McKeegan D.E.F. (2009). Public opinion on UK milk marketing and dairy cow welfare. Anim. Welf..

[37] Degeling C., Johnson J. (2015). Citizens, consumers and animals: What role do experts assign to public values in establishing animal welfare standards?. J. Agric. Environ. Ethics.

[38] Cornish A., Raubenheimer D., McGreevy P. (2016). What we know about the public’s level of concern for farm animal welfare in food production in developed countries. Animals.

[39] Hemsworth P.H., Mellor D.J., Cronin G.M., Tilbrook A.J. (2015). Scientific assessment of animal welfare. N. Z. Vet. J..

[40] Fisher M.W. (2009). Defining animal welfare—Does consistency matter?. N. Z. Vet. J..

[41] Vanhonacker F., Verbeke W., Van Poucke E., Tuyttens F.A.M. (2008). Do citizens and farmers interpret the concept of farm animal welfare differently?. Livest. Sci..

[42] Broom D.M. (1986). Indicators of poor welfare. Brit. Vet. J..

[43] Fraser A.F., Broom D.M. (1990). Farm Animal Behaviour and Welfare.

[44] Sumner L.W. (1996). Welfare, Happiness, and Ethics.

[45] Dawkins M.S. (1988). Behavioral deprivation: A central problem in animal welfare. Appl. Anim. Behav. Sci..

[46] Duncan I.J.H., Petherick J.C. (1991). The implications of cognitive processes for animal welfare. J. Anim. Sci..

[47] Sandøe P. (1996). Animal and human welfare—Are they the same kind of thing?. Acta Agric. Scand. Anim. Sci..

[48] Fraser D. (2003). Assessing animal welfare at the farm and group level: The interplay of science and values. Anim. Welf..

[49] Vanhonacker F., Verbeke W. (2014). Public and consumer policies for higher welfare food products: Challenges and opportunities. J. Agric. Environ. Ethics.

[50] Broom M.D., Broom M.D., Gil G.R. (2007). Introducción: conceptos sobre la protección y el bienestar animal que incluyen las obligaciones y los derecho. Bienestar Animal.

[51] Bracke M.B.M., Spruijt B.M., Metz J.H.M. (1999). Overall animal welfare assessment reviewed. Part 1: Is it possible?. Neth. J. Agr. Sci..

[52] Botreau R., Veissier I., Butterworth A., Bracke M.B.M., Keeling L.J. (2007). Definition of criteria for overall assessment of animal welfare. Anim. Welf..

[53] Vapnek J.C., Chapman M. (2010). Legislative and Regulatory Options for Animal Welfare.

[54] Mellor D.J. (2016). Updating animal welfare thinking: Moving beyond the “Five Freedoms” towards “A Life Worth Living”. Animals.

[55] Green T.C., Mellor D.J. (2011). Extending ideas about animal welfare assessment to include ‘quality of life’ and related concepts. N. Z. Vet. J..

[56] Lassen J., Sandoe P., Forkman B. (2006). Happy pigs are dirty! Conflicting perspectives on animal welfare. Livest. Sci..

[57] Webb E.C. (2013). The ethics of meat production and quality—A South African perspective. S. Afr. J. Anim. Sci..

[58] Sandøe P., Christiansen S.B., Appleby M.C. (2003). Farm animal welfare: The interaction of ethical questions and animal welfare science. Anim. Welf..

[59] Sandøe P., Corr S.A., Lund T.B., Forkman B. (2019). Aggregating animal welfare indicators: Can it be done in a transparent and ethically robust way?. Anim. Welf..

[60] Weary D.M., Robbins J.A. (2019). Understanding the multiple conceptions of animal welfare. Anim. Welf..

[61] Grunert K.G. (2006). Future trends and consumer lifestyles with regard to meat consumption. Meat Sci..

[62] Clark B., Stewart G.B., Panzone L.A., Kyriazakis I., Frewer L.J. (2017). Citizens, consumers and farm animal welfare: A meta-analysis of willingness-to-pay studies. Food Policy.

[63] de Graaf S., Van Loo E.J., Bijttebier J., Vanhonacker F., Lauwers L., Tuyttens F.A.M., Verbeke W. (2016). Determinants of consumer intention to purchase animal-friendly milk. J. Dairy Sci..

[64] Eagly A.H., Chaiken S. (1993). The psychology of Attitudes.

[65] Fransson N., Garling T. (1999). Environmental concern: Conceptual definitions, measurement methods, and research findings. J. Environ. Psychol..

[66] Brom F.W.A. (2000). Food, consumer concerns, and trust: Food ethics for a globalizing market. J. Agric. Environ. Ethics.

[67] Verbeke W., Viaene J. (1999). Beliefs, attitude and behaviour towards fresh meat consumption in Belgium: Empirical evidence from a consumer survey. Food Qual. Prefer..

[68] Harper G., Henson S. (2001). Consumer Concerns about Animal Welfare and the Impact on Food Choice.

[69] Ajzen I. (1991). The theory of planned behavior. Organ. Behav. Hum. Decis. Process..

[70] McInerney J. (2004). Animal Welfare, Economics and Policy.

[71] Miele M., Evans A. (2010). When foods become animals: Ruminations on ethics and responsibility in care-full practices of consumption. Ethics Place Environ..

[72] Blokhuis H.J., Keeling L.J., Gavinelli A., Serratosa J. (2008). Animal welfare’s impact on the food chain. Trends Food Sci. Technol..

[73] McEachern M.G., Schröder M.J.A., Willock J., Whitelock J., Mason R. (2007). Exploring ethical brand extensions and consumer buying behaviour: The RSPCA and the “Freedom Food” brand. J. Prod. Brand Manag..

[74] Miele M. (2010). Report Concerning Consumer Perceptions and Attitudes towards Farm Animal Welfare.

[75] Boogaard B.K., Bock B.B., Oosting S.J., Wiskerke J.S.C., van der Zijpp A.J. (2011). Social acceptance of dairy farming: The ambivalence between the two Faces of modernity. J. Agric. Environ. Ethics.

[76] Blandford D., Bureau J.C., Fulponi L., Henson S., Krissoff B., Bohman M., Caswell J.A. (2002). Potential implications of animal welfare concerns and public policies in industrialized countries for international trade. Global Food Trade and Consumer Demand for Quality.

[77] de Jonge J., van Trijp H.C.M. (2013). Meeting heterogeneity in consumer demand for animal welfare: A reflection on existing knowledge and implications for the meat sector. J. Agric. Environ. Ethics.

[78] Bernues A., Olaizola A., Corcoran K. (2003). Extrinsic attributes of red meat as indicators of quality in Europe: An application for market segmentation. Food Qual. Prefer..

[79] Gellynck X., Verbeke W., Vermeire B. (2006). Pathways to increase consumer trust in meat as a safe and wholesome food. Meat Sci..

[80] Hoffman L.C., Wiklund E. (2006). Game and venison—Meat for the modern consumer. Meat Sci..

[81] McEachern M.G., Willock J. (2004). Producers and consumers of organic meat: A focus on attitudes and motivations. Br. Food J..

[82] Ingenbleek P.T.M., Immink V.M. (2011). Consumer decision-making for animal-friendly products: Synthesis and implications. Anim. Welf..

[83] Cardoso C.S., Hoetzel M.J., Weary D.M., Robbins J.A., von Keyserlingk M.A.G. (2016). Imagining the ideal dairy farm. J. Dairy Sci..

[84] Yiridoe E.K., Bonti-Ankomah S., Martin R.C. (2005). Comparison of consumer perceptions and preference toward organic versus conventionally produced foods: A review and update of the literature. Renew. Agr. Food. Syst..

[85] Martelli G. (2009). Consumers’ perception of farm animal welfare: An Italian and European perspective. Ital. J. Anim. Sci..

[86] Bennett R., Kehlbacher A., Balcombe K. (2012). A method for the economic valuation of animal welfare benefits using a single welfare score. Anim. Welf..

[87] Moran D., McVittie A. (2008). Estimation of the value the public places on regulations to improve broiler welfare. Anim. Welf..

[88] Grunert K.G., Sonntag W.I., Glanz-Chanos V., Forum S. (2018). Consumer interest in environmental impact, safety, health and animal welfare aspects of modern pig production: Results of a cross-national choice experiment. Meat Sci..

[89] Harper G.C., Makatouni A. (2002). Consumer perception of organic food production and farm animal welfare. Br. Food J..

[90] Palupi E., Jayanegara A., Ploeger A., Kahl J. (2012). Comparison of nutritional quality between conventional and organic dairy products: A meta-analysis. J. Sci. Food Agric..

[91] Buller H., Morris C. (2003). Farm animal welfare: A new repertoire of nature-society relations or modernism re-embedded?. Sociol. Ruralis.

[92] Vanhonacker F., Verbeke W. (2009). Buying higher welfare poultry products? Profiling Flemish consumers who do and do not. Poult. Sci..

[93] Troy D.J., Kerry J.P. (2010). Consumer perception and the role of science in the meat industry. Meat Sci..

[94] Wood J.D., Enser M., Fisher A.V., Nute G.R., Richardson R.I., Sheard P.R. (1999). Manipulating meat quality and composition. Proc. Nutr. Soc..

[95] Fearne A., Hornibrook S., Dedman S. (2001). The management of perceived risk in the food supply chain: A comparative study of retailer-led beef quality assurance schemes in Germany and Italy. Int. Food Agribus. Man..

[96] Muller Lindenlauf M., Deittert C., Kopke U. (2010). Assessment of environmental effects; animal welfare and milk quality among organic dairy farms. Livest. Sci..

[97] de Jonge J., van Trijp H. (2014). Heterogeneity in consumer perceptions of the animal friendliness of broiler production systems. Food Policy.

[98] Marks and Spencer Animal Welfare 2015. http://corporate.marksandspencer.com/plan-a/policies/our-policies/food/animal-welfare.

[99] Lagerkvist C.J., Hess S. (2011). A meta-analysis of consumer willingness to pay for farm animal welfare. Eur. Rev. Agric. Econ..

[100] Sepulveda W.S., Maza M.T., Pardos L. (2011). Aspects of quality related to the consumption and production of lamb meat. Consumers versus producers. Meat Sci..

[101] Toma L., Stott A.W., Revored Giha C., Kupiec Teahan B. (2012). Consumers and animal welfare. A comparison between European Union countries. Appetite.

[102] Phillips C.J.C., Izmirli S., Aldavood S.J., Alonso M., Choe B.L., Hanlon A., Handziska A., Illmann G., Keeling L., Kennedy M. (2012). Students’ attitudes to animal welfare and rights in Europe and Asia. Anim. Welf..

[103] Knight S., Barnett L. (2008). Justifying attitudes toward animal use: A qualitative study of people’s views and beliefs. Anthrozoos.

[104] Miranda de la Lama G.C., Sepulveda W.S., Villarroel M., Maria G.A. (2013). Attitudes of meat retailers to animal welfare in Spain. Meat Sci..

[105] Makdisi F., Marggraf R. (2011). Consumer willingness-to-pay for farm animal welfare in Germany. The case of broiler. Proceedings of the 51st Annual Conference.

[106] Harvey D., Hubbard C. (2013). Reconsidering the political economy of farm animal welfare: An anatomy of market failure. Food Policy.

[107] Glass C.A., Hutchinson W.G., Beattie V.E. (2005). Measuring the value to the public of pig welfare improvements: A contingent valuation approach. Anim. Welf..

[108] Nocella G., Hubbard L., Scarpa R. (2010). Farm animal welfare, consumer willingness to pay and trust: Results of a cross-national survey. Appl. Econ. Perspect. Policy.

[109] Vanhonacker F., Van Poucke E., Tuyttens F., Verbeke W. (2010). Citizens’ views on farm animal welfare and related information provision: Exploratory insights from Flanders, Belgium. J. Agric. Environ. Ethics.

[110] Grandin T. (2014). Animal welfare and society concerns finding the missing link. Meat Sci..

[111] Jensen H.H. (2006). Consumer issues and demand. The magazine of food, farm, and resource issues. Choices.

[112] Boogaard B.K., Oosting S.J., Bock B.B. (2006). Elements of societal perception of farm animal welfare: A quantitative study in The Netherlands. Livest. Sci..

[113] Kjærnes U., Lavik R., Kjærnes U., Miele M., Roex J. (2007). Farm animal welfare and food consumption practices: Results from surveys in seven countries. Attitudes of Consumers, Retaliers and Producers to Farm Animal Welfare.

[114] Te-Velde H., Aarts N., Van Woerkum C. (2002). Dealing with ambivalence: Farmers’ and consumers’ perceptions of animal welfare in livestock breeding. J. Agric. Environ. Ethics.

[115] Buller H., Cesar C. (2007). Eating well, eating fare: Farm animal welfare in France. Int. J. Sociol. Food Agric..

[116] Abidoye B.O., Bulut H., Lawrence J., Mennecke B., Townsend A.M. (2011). US consumers’ valuation of quality attributes in beef products. J. Agric. Appl. Econ..

[117] Schröder M.J.A., McEachern M.G. (2004). Consumer value conflicts surrounding ethical food purchase decisions: A focus on animal welfare. Int. J. Consum. Stud..

[118] Kunst J.R., Hohle S.M. (2016). Meat eaters by dissociation: How we present, prepare and talk about meat increases willingness to eat meat by reducing empathy and disgust. Appetite.

[119] Berry H., McEachern M.G., Harrison R.N.T., Shaw D.S. (2005). Informing ethical consumers. The Ethical Consumers.

[120] Frewer L.J., Kole A., Van De Kroon S.M.A., De Lauwere C. (2005). Consumer attitudes towards the development of animal-friendly husbandry systems. J. Agric. Environ. Ethics.

[121] Mayfield L.E., Bennett R.M., Tranter R.B., Wooldridge M.J. (2007). Consumption of welfare-friendly food products in Great Britain, Italy and Sweden, and how it may be influenced by consumer attitudes to, and behaviour towards, animal welfare attributes. Int. J. Sociol. Food Agric..

[122] Veissier I., Butterworth A., Bock B., Roe E. (2008). European approaches to ensure good animal welfare. Appl. Anim. Behav. Sci..

[123] Barnett J.L. (2007). Effects of confinement and research needs to underpin welfare standards. J. Vet. Behav..

[124] Napolitano F., Girolami A., Braghieri A. (2010). Consumer liking and willingness to pay for high welfare animal-based products. Trends Food Sci. Technol..

[125] Toma L., McVittie A., Hubbard C., Stott A.W. (2011). A structural equation model of the factors influencing British consumers’ behaviour toward animal welfare. J. Food Prod. Mark..

[126] Gracia A., Loureiro M.L., Nayga R.M. Valuing animal welfare labels with experimental auctions: What do we learn from consumers?. Proceedings of the International Association of Agricultural Economists Conference.

